# Development and Optimization of a Multilayer Rat Purkinje Neuron Culture

**DOI:** 10.1007/s12311-022-01510-4

**Published:** 2023-01-10

**Authors:** Ida Margrethe Uggerud, Torbjørn Kråkenes, Hirokazu Hirai, Christian Alexander Vedeler, Manja Schubert

**Affiliations:** 1 Department of Neurology, Haukeland University Hospital, 5021 Bergen, Norway; 2 Department of Clinical Medicine (K1), University of Bergen, 5021 Bergen, Norway; 3 Department of Neurophysiology &, Neural Repair, Gunma University Graduate School of Medicine, Maebashi, Gunma 371-8511 Japan; 4 Neuro-SysMed - Centre of Excellence for Experimental Therapy in Neurology, Departments of Neurology and Clinical Medicine, 5021 Bergen, Norway; 5 Institute of Anatomy, Faculty of Medicine Carl Gustav Carus, 01307 Dresden, Germany

**Keywords:** Ex vivo culture system; Rat Purkinje neurons, Three-dimensional cerebellar network

## Abstract

Elucidation of the mechanisms involved in neurodegenerative diseases of the cerebellum has been hampered by the lack of robust single cell models to study Purkinje neurons and replicate at the same time in vivo features. Cerebellar Purkinje neurons are difficult to grow in dispersed cell culture, and only limited work has been done using rat cells. We developed a refined protocol for growing rat Purkinje neurons from embryonic and postnatal tissue ex vivo that results in well-developed, mature, functional, and synaptically active neurons. The rat Purkinje neurons generated are responsive to paracrine factors and genetic manipulation, allowing great experimental flexibility at the single-cell level. This ex vivo model can be used to investigate disease mechanisms that disturb Purkinje neuron morphology, function, and communication in high- and low-throughput screening formats.

## Introduction

Neuronal culture systems enable the study of basic principles of the nervous system and disease mechanisms. Ex vivo studies of the neuronal network and its alterations during disease are often done in organotypic slice cultures, but single-cell analyses, genetic modifications, and long-term studies are difficult or impossible with this ex vivo approach. Dissociated neuronal cultures allow manipulation and observation of neurons at the single-cell level; however, the quality and survival of the cultured cells depend on several factors: these include animal species, age of tissue used to derive single cells, the surface onto which single cells are seeded and cultured, and co-factors that drive neuronal growth and development [1–5]. To date, the majority of successful Purkinje neuron culture models have used embryonic mouse cerebelli [3, 5]. It has proven more difficult to culture and transgenically manipulate cells from rats [1, 2, 4, 6], but rats are physiologically, immunologically, genetically, and morphologically closer to humans than are mice [6, 7]. Furthermore, outbred or transgenic rat models [8–10] mimic human neurodegenerative disease mechanisms and progressions more closely than mouse models do [11, 12].

Neurodegeneration generally occurs in the adult or aged human brain; therefore, a dissociated culture system derived from mature rather than embryonic tissue is desirable in investigation of the mechanisms behind pathological processes. Nonetheless, previous attempts to culture functional dissociated neurons from late postnatal tissue have largely been unsuccessful [1]. Our goal was, therefore, to develop a culture protocol that provided well-developed, mature, functional, and synaptically active rat Purkinje neurons independent of tissue age that resulted in maximal experimental flexibility and the potential for high-throughput screening. We discovered four essential factors for success: use of a three-dimensional growth structure, pH stability, co-factor supplementation and not using tissue after postnatal day 10. Using more mature or adult tissue at postnatal day 14, 28, 60, and 180 did not, yield surviving Purkinje neurons.

## Materials and Methods

### Neuronal Culture Preparation

All procedures were performed according to the National Institutes of Health Guidelines for the Care and Use of Laboratory Animals Norway (FOTS 20,135,149/20157494/20170001). Wistar Hannover GLAST rat pups (*n* = 328), embryonic day 18 (E18) to postnatal day 10 (P10), were used for neuronal culture preparation. Briefly, following anaesthesia and decapitation, the brains were rapidly transferred into preparation solution of ice cold EBSS (Gibco, #24,010,043) solution containing 0.5% glucose (Sigma, #G8769) and 10 mM HEPES (Gibco, #15,630,056). Under a dissection microscope, the meninges and medulla oblongata were carefully removed, and the cerebellum was separated from the pons and the midbrain. Depending on the culture, either only the cerebellum or the cerebellum including pons was transferred to a 15-mL tube containing 20 U/mL papain (Worthington, #LK003178) dissolved in preparation solution and warmed up to 36 °C. The tube containing the cerebellar tissue was placed into the incubator for 15 min at 36 °C with occasionally swirling to digest the tissue. The papain solution was carefully removed with a fire-polished Pasteur pipette, and the digestion reaction was stopped by addition of stop media (advanced DMEM/F12 solution (Gibco, #12,634,010) containing 0.5% glucose (Sigma, #G8769) and 10% foetal bovine serum (FBS, Gibco, #10,500,064)), pre-warmed to 36 °C. After 5 min of deactivation, the stop media was removed and 250 µL growth media containing 10% FBS per cerebellum was added. The tissue/media suspension was pipetted up and down with a fire-polished Pasteur pipette at least 100 times until cells were separated.

### Support Cell Layer

To grow the support cell layer, first 375.000 cells per mL were isolated from the cerebellum and pons of embryonic (E18) or postnatal day 0 rat pups and seeded on coverslips pre-coated with poly-d-lysine (PDL; Neuvitro: #GG-12–1.5-PDL, 24 well, 500 µL/well; #GG-18–1.5-PDL, 12 well, 1 mL/well; #GG-25–1.5-laminin, 6 well, 2 mL/well). The support cell layer cultures were maintained in 6-, 12-, or 24-well plates in a medium based on the Furuya protocol [2] with modifications. The growth medium was made of 45% advanced DMEM/F12 solution (Gibco, #126,340,010), 45% NBM solution (Miltenyi Biotec, #130–093-570), 1.5% B-27 serum-free supplement (Gibco, #17,504,044), 1.5% NB-21 serum-free supplement (Miltenyi Biotec, #130–093-566), 1% sodium pyruvate (Invitrogen, #11,360,088), 1% heat-inactivated FBS (Invitrogen, #10,500,064), 2% Glutamax (Gibco, #35,050,038), 5 mg/mL D-glucose, and 10 mM HEPES (Invitrogen, #15,630,056). The media volume of each well depended on the plate size: 1500 µL for 6-well plates, 750 µL for 12-well plates, and 400 µL for 24-well plates. Half of the culture medium was replaced every 7 days.

### Purkinje Neuron Layer

Neuronal cells can survive only within a narrow pH range. The effects of the extracellular and intracellular pH on numerous enzymes and channels is an extensive subject which is beyond the scope of this study. However, it has been found that acidification can inhibit important classes of synaptic channels such as VGCC [13], whereas alkalinisation increases neuronal activity [14]. Purkinje neurons and unipolar brush cells of the cerebellum are rich in acid-sensitive ion channels [15] and rich in proteins that control calcium and thereby activity. We have found that a neutral pH, in the slightly acid range of 6.8 to 7.0, was essential for cell survival of newly plated Purkinje neurons, and therefore, the support cell layer culture was fed 24 h prior to addition of the Purkinje neuron layer to ensure a pH in this range. E18- and P0-derived Purkinje neuron cultures were prepared by seeding 500.000 cells per mL from the vermis and the flocculus of the cerebellum onto support cell layers of different in vitro ages. The P10-derived Purkinje neuron culture was prepared by seeding 750.000 cells per mL from only the vermis of the cerebellum onto the support cell layers of different in vitro ages. Depending on the plate, a different volume of single-cell suspension was added to the support cell culture per well: 500 µL for 6-well plates, 250 µL for 12-well plates, and 100 µL for 24-well plates. The support cell layer growth media was supplemented with insulin (Invitrogen, #12,585,014; 1:250, stock 4 mg/mL), progesterone (Sigma, #P8783, 1:2000, stock 80 mM), insulin-like growth factor 1 (IGF1; Promokine, #E-60840, 1:40,000, stock 1 µg/µL), and protein kinase C inhibitor K252a (Alomone, # K-150; IC_50_ 25 nM). In cultures that were maintained for more than 28 days in vitro, the IGF1 and progesterone concentrations were reduced to 10 ng/mL and 20 µM, respectively. K252a was supplemented for 21 days before the washout process started, its optimal concentration was experimentally determined for each culture type. In the first 10 days of culturing, the K252a concentration was 5 nM for E18-derived Purkinje neuron layers, 10 nM for P0-derived Purkinje neuron layers, and 25 nM for P10-derived Purkinje neuron layers. After 10 days, the K252a concentration was increased to 25 nM for all Purkinje neuron layers until day 21 in vitro. After 21 days in vitro (*DIV*), the washout phase of K252a was implemented by replacing half of the media with media that did not contain K252a. Half of the culture medium was replaced every 3.5 days for 6-well plates and every 2 days for 12- and 24-well plates. The experiments that determined different culture parameters and their impact on the Purkinje neuron yield were randomly performed, three to six times per experimental setting with five independent repeats for each group and condition.

### Lentiviral Gene Editing

The full-length L7 promoter region (1005 bp) [16, 17] was custom cloned by SBI System Bioscience into construct pCDH-L7-MCS-copGFP (#CS970S-1), and viral particles with a yield of 2.24 × 10^9 infectious units per µL were produced. Two viral transduction approaches were tested. First, freshly prepared cells of E18 or P0 rat cerebellum (suspended in growth media containing no serum) were incubated for 10 min at 36 °C with 1.22 × 10^6 viral particles per mL cell suspension and were then seeded onto the support cell layer culture. To grow the transduced neurons, either 12-well plates containing coverslips or 35-mm µ-dishes for live-cell imaging (Ibidi, #80,136) were used. Media was replaced after 3 days, and transfection efficiency was evaluated by live-cell microscopy 24 h post transfection, and then daily until 21 *DIV.* At 21 *DIV*, the scan interval was changed from daily to every 3 or 4 days for cultures of E18 or P0 rat cerebellum, respectively, until 169 *DIV*. In the second experimental approach, the lentiviral transduction was performed on neurons in culture. Cells were fed on 15 *DIV* and 29 *DIV*, for cultures of E18 or P0 rat cerebellum, respectively, and 1 day later 2.5 × 10^6 viral particles per mL were applied to the culture media.

The neuronal morphology of GFP-expressing Purkinje neurons was analysed by capturing 10 independent 3 × 3 tile scans (Zyla camera configuration: 2048 × 2048; objectives: CFI Plan Apochromat Lambda dry objective 10 (NA 0.45, pixel size 603 nm) or dry objective 20 (NA 0.75, pixel size 301 nm)) on an Andor Dragonfly microscope system (Oxford Instruments). The viral transduction of the cultures was repeated three times.

### Immunohistochemical Characterisation of Cell Types

Cultures were probed extensively by immunofluorescence microscopy to characterize their component cell types (see Table 1). Table 1 provides a detailed list of the used antibodies, indexing cell-specificity, concentration, antibody species, and staining conditions. Culture were washed with pre-warmed 0.1 M PBS (1xPBS; Gibco, #70,013,016) and fixed with 1.5–4% paraformaldehyde (pH 6–7.2; ThermoScientific, #28,908) containing 0.5% sucrose for 15 min at 36 °C. Tris-based or citric acid-based heat-induced antigen retrieval (pH 9 and pH 6, respectively; 45 min, 85 °C) [18] was performed for some targets (Table 1). The cultures were quenched with 1xPBS containing 50 mM NH_4_Cl (PBS_N_), permeabilised with 0.2% Triton X-100 (Sigma, #T9284) in PBS_N_ (5 min, 36 °C), rinsed with PBS_N_ containing 0.5% cold water fish gelatine (Sigma, #G7041; PBS_NG_, 3 × 15 min), and incubated with primary antibodies overnight at 4 °C in PBS_NG_ containing 10% Sea Block (Thermo Scientific, #37,527), 0.05% Triton X-100, and 100 μM glycine (Sigma, #G7126). The coverslips were rinsed with PBS_NG_ (three times, 20 min) and incubated with highly cross-absorbed donkey secondary antibodies conjugated to CF™ 488/594/647 dye (Biotium, #20,014, #20,115, #20,046, #20,015, #20,152, #20,047, #20,074, #20,075, #20,169, #20,170; 1:400) for 2 h at room temperature in PBS_NG_ containing 2.5% Sea Block. To remove unbound secondary antibody, coverslips were rinsed with PBS_N_ (three times, 20 min), and briefly dipped into MilliQ water before mounting onto microscope glass using Prolong™ Glass Antifade Reagent (Invitrogen, #P36981). The mounted cultures were kept 2 days at room temperature in the dark, followed by long-term storage at 4 °C until imaging.

**Table 1:** Primary antibodies. The signal to noise ratio for the antibodies were evaluated for the following conditions: 4% PFA at pH 7.2 diluted in 100 mM PBS; 1.5% PFA at pH 6 diluted in 100 mM natrium acetate buffer (NaAcB)); without heat-induced antigen retrieval (HIAGR); and with HIAGR either TRIS-based (pH 9) or citric acid-based (pH 6). The best conditions for each used antibody are described below

Antibody	Species	Company	Cat. No	LOT No	RRID	Dilution [µg/mL]	PFA fixation	HIAGR	Marker
α-synuclein	Chicken	EnCorBio	CPCA-SNCA	71,113	AB_2572385	1.0	1.5%; pH 6; NaAcB	No	Pre-synapse, granule and unipolar brush cells /PNs
Bassoon	Chicken	SYSY	141,016	141,016/1–1	AB_2661779	Serum 1:500	4%, pH 7.2; PBS	No	Pre-synapse; Golgi/granule cells, or basket cells/PNs
Calbindin	Guinea pig	SYSY	214,005	214,005/1–5	AB_2619902	0.5	4%, pH 7.2; PBS	No	Purkinje neurons
	Chicken	SYSY	214,006	214,006/1–3	AB_2619903	Serum 1:750	4%, pH 7.2; PBS	No	Purkinje neurons
Calretinin	Chicken	SYSY	214,106	214,106/2	AB_2619909	Serum 1:500	4%, pH 7.2; PBS	No	Unipolar-brush cells
CNP1	Rabbit	SYSY	355,003	355,003/1–2	AB_2620112	1.0	4%, pH 7.2; PBS	No	Oligodendrocytes
GABA_A_Rα6	Rabbit	SYSY	224,603	224,603/3	AB_2619945	5.0	4%, pH 7.2; PBS	pH 9	Granule cells
GAD65	Mouse	BD Bio-science	559,931	4,283,665	AB_397380	2.5	1.5%; pH 6; NaAcB	No	Pre-synapse, stellate and basket cells/PNs
GlyT2	Guinea pig	SYSY	272,004	27,004/2	AB_2619998	Serum 1:250	4%, pH 7.2; PBS	pH 6	Golgi cells; Lugaro cells
IBA1	Rabbit	EnCorBio	RPCA-IBA1	266_100517	AB_2722747	1.0	4%, pH 7.2; PBS	No	microglia
mGluR1	Guinea pig	FRONTIER	2,571,801		AB_2571801	2.5	1.5%; pH 6; NaAcB	No	PNs, Lugaro cells
Neurogranin	Rabbit	SYSY	357,003	357,003/1	AB_2620115	2.5	4%, pH 7.2; PBS	No	Golgi cells
Parvalbumin	Guinea pig	SYSY	195,004	195,004/1–21	AB_2156476	Serum 1:500	4%, pH 7.2; PBS	No	PNs, basket and stellate cells
PCP2	Rabbit	Takara	M194	1AFXJ002.0		1.0	4%, pH 7.2; PBS	No	Purkinje neurons
Peripherin	Rabbit	EnCor Bio	RPCA-Peri	0208_070316	AB_2572375	0.5	4%, pH 7.2; PBS	No	Mossy and climbing fibers
PSD95	Mouse	Neuro mab	75–028	455.7JD.22G	AB_2292909	5.0	1.5%; pH 6; NaAcB	No	Post-synapse
Synapsin 1/2	Chicken	SYSY	106,006	106,006/1–4	AB_2622240	Serum 1:500	1.5%; pH 6; NaAcB	No	Pre-synapse
VGCC-PQ α-1A	Guinea pig	SYSY	152,205	152,205/3	AB_2619842	4.0	1.5%; pH 6; NaAcB	No	Purkinje neuron synapse
VGluT2	Guinea pig	SYSY	135,404	135,404/2–32	AB_887884	Serum 1:500	4%, pH 7.2; PBS	pH 6	Mossy and climbing fibers

*EnCorBio*, EnCor Biotechnology; *SYSY*, synaptic systems; *PN*, Purkinje neuron

### Purkinje Neuron Counting and Imaging

Purkinje neurons were visualized by immunohistochemical staining for calbindin (CALB1) or Purkinje cell protein 2 (PCP2) and counted manually by blinded investigators using a Leitz Diaplan fluorescence microscope (equipped with CoolLED pE-300white lamp). In addition, 10 Z-stack images were taken per coverslip (5 independent fields of view) on an Andor Dragonfly microscope system (Zyla camera configuration: 2048 × 2048; CFI Plan Apochromat Lambda objectives: S LWD water 40 × NA 1.14 (pixel size 151 nm), oil 60 × NA 1.20 (pixel size 103 nm), oil CFI SR HP Apo TIRF 100 × NA 1.49 (pixel size 60 nm)). The images were superimposed with the Fusion software (Oxford Instruments).

### Dendritic Tree Branch Analysis

The dendritic development (length and order) of the Purkinje neurons was evaluated by analysing 10 Purkinje neurons per coverslip in 10 independent experiments using the ImageJ plugin Simple_Neurit_Tracer (SNT; Neuroanatomy) [19]. This free software plugin is available at http://fiji.sc/Simple_Neurite_Tracer. The plugin allows manually selection of points along tubular structures in confocal microscope z-stack images to trace the dendritic skeleton of neurons. The program marks the path between two branch points by determining the highest fluorophore emission intensity. This semi-automated process ensures that the right paths are chosen and traced. Each dendritic tree was evaluated with branching grades (orders) according to the Fujishima protocol [20].

The branch orders for each analyzed Purkinje neuron were determined as follows: Microscope Leica files (.lif) were converted to TIFF files and opened with the Fiji SNT plugin. The reconstruction of Purkinje neuron dendritic branching started with tracing of the primary dendrites by selecting the soma as the starting point and selecting the point of intersection between primary and secondary dendrite segments. Tracing non-primary dendrites was done by selecting a new point on the next dendrite intersection or the ending point of the dendrite. This was performed for the whole dendritic arbor of each Purkinje neuron. The data were exported to Excel where the average length of dendrites of the same order, as well as the number of segments of the same order, was evaluated for each analyzed Purkinje neuron.

### 3D Representation of Dendrites and Synapses

3D surface visualization of synapses was performed using Oxford Instruments analysis software IMARIS 9.3.1 (Bitplane) and the filament tracer tool [21]. The “surface” tool was applied to make a solid filled surface representation of the Purkinje neurons through the CALB1 or PCP2 staining, with the background subtraction option enabled. A threshold was selected that demarcated the neuron structure accurately while excluding background. For the synapse analyses, a 600 × 800 µm selection box was placed around the dendrite in each image, and surfaces were created for synapses within the selection box. The same threshold settings were used across all images, and individual surface data from each dendrite were exported for the 3D representation of the synapses.

### Micro-electrode Array Recordings

E18 culture at a concentration of 500.000 cells per mL was plated onto 24-well plates of the Multiwell-MEA-System pre-coated with PDL (Multichannel Systems, Reutlingen). Each well contained 12 PEDOT-coated gold micro-electrodes (30 µm diameter, 300 µm space, 3 × 4 geometrical layout) on a glass base (#890,850, 24W300/30G-288). The amplifier (data resolution: 24 bit; bandwidth: 0.1 Hz to 10 kHz, modifiable via software; default 1 Hz to 3.5 kHz; sampling frequency per channel: 50 kHz or lower, software controlled; input voltage range: ± 2500 mV), stimulator (current stimulation: max. ± 1 mA; voltage stimulation: max. ± 10 V; stimulation pattern: pulse or burst stimulation sites freely selectable), and heating element (regulation: ± 0.1 °C) were integrated into the Multiwell-MEA headstage, which was driven by the MCS-Interface Board 3.0 Multiboot. The Multiwell recording platform was covered by a mini incubator (5% CO_2_; balanced air). Electrophysiological signals were acquired at a sampling rate of 20 kHz (Multiwell-Screen software). Plates were tested for spontaneous activity every second day from 5 *DIV* on. Raw voltage traces were recorded for 120 s to calculate spike rate and burst activity using offline MCS-Multiwell-Analyzer. The spontaneous activity of the culture was evaluated for two different conditions. In the first condition, cultures were maintained in Purkinje neuron culture media (45% advanced DMEM/F12 solution, 45% NBM solution, 1.5% B-27 serum-free supplement, 1.5% NB-21 serum-free supplement, 1% sodium pyruvate, 1% heat-inactivated FBS, 2% GlutaMAX, 5 mg/mL D-glucose, 10 mM HEPES, 16 µg/mL insulin, 25 ng/mL IGF1, 40 µM progesterone, 5 nM K252a) for 63 days. In the second condition, the Purkinje neuron culture media was exchanged to organotypic slice culture media [22] (30% advanced DMEM/F12 solution, 20% MEM solution (Gibco, #41,090,028), 25% EBSS solution (Gibco, #24,010,043), 25% heat-inactivated horse serum (Sigma, #H1138), 2% GlutaMAX, 5 mg/ml D-glucose, and 2% B-27 serum-free supplement) on the 28 *DIV*, and cultures were monitored for an additional 45 days.

### Statistical Analysis

All conditions were tested in triplicate or quadruplicate, if not otherwise indicated. Data analysis and calculations were performed using the software Excel 2016 and Graph Pad Prism 7.0. Data are presented as means ± SEM. Statistical significance was determined using the non-parametric two-tailed paired Mann–Whitney’s *U* test. The level of significance is indicated with asterisks: *p < 0.05; **p < 0.01; ***p < 0.001.

## Results

### Rat Purkinje Neuron Culture: Monolayer Versus Multilayer Culture

**Fig. 1:**
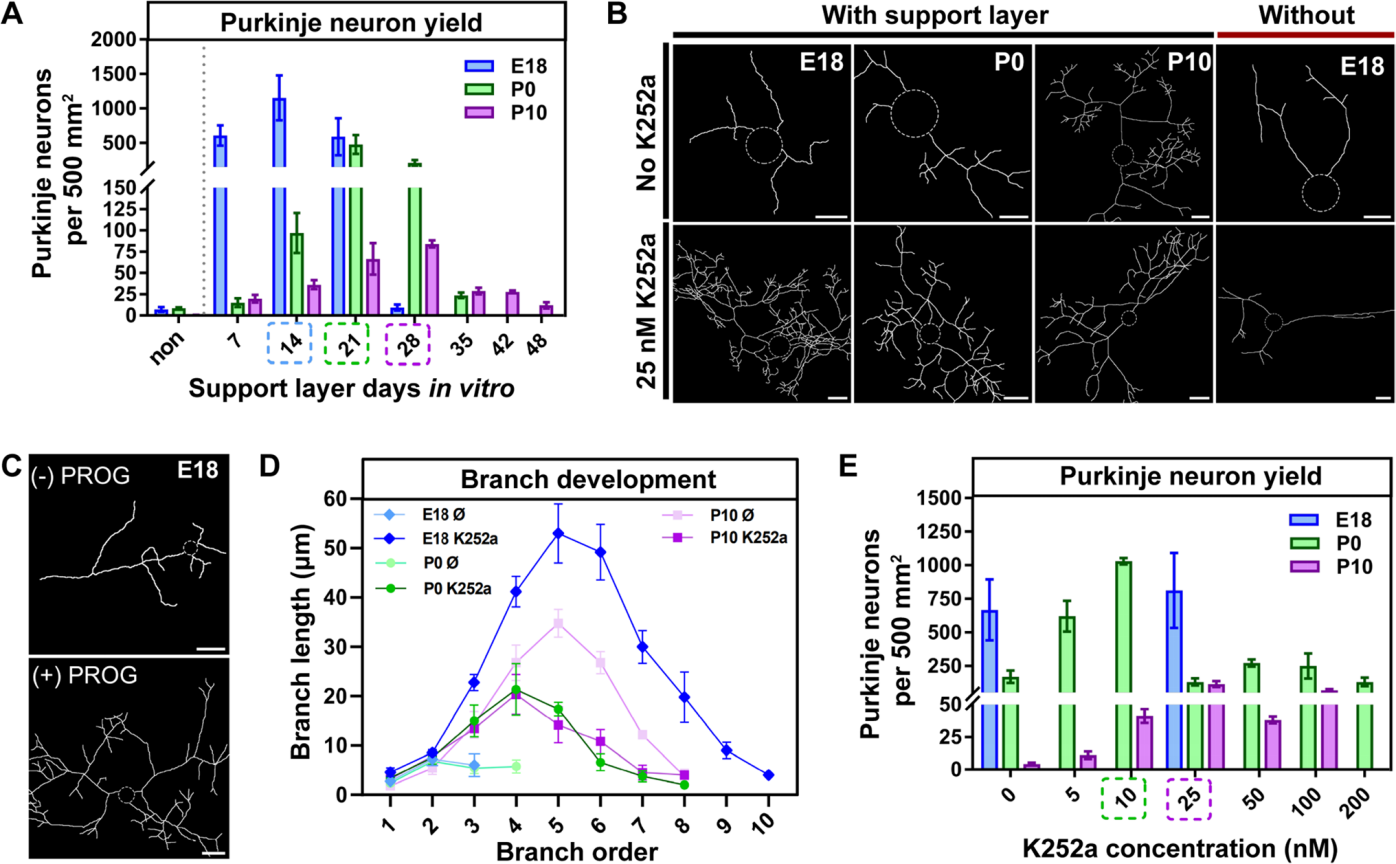
Evaluation of age- and co-factor-dependence of rat Purkinje neuron culture at 21 days in vitro. **A** Relationship of Purkinje neuron yield to in vitro age of the support cell layer (*DIV* 7 to 48) for E18-, P0-, and P10-derived Purkinje neurons. **B** Representative skeletons of E18-, P0-, and P10-derived Purkinje neurons with and without PKC antagonist K252a and of E18-derived cultures grown on support layer or not. Scale bar: 20 µm. **C** Representative skeletons of E18-derived Purkinje neurons without and with 40 µM progesterone. Scale bar: 20 µm. **D** Plots of dendritic branch length versus branch order for Purkinje neurons derived from E18, P0, and P10 tissue without and with 25 nM K252a to modulate PKC activity. **E** Relationship of Purkinje neuron yield to concentration of K252a for E18-, P0-, and P10-derived Purkinje neurons

We first attempted to grow Purkinje neurons directly as a monolayer on glass coverslips coated with PDL and the extracellular matrix protein laminin [1, 2]. This approach failed. The survival rate of Purkinje neurons per coverslip was low or even declined to zero after 21 *DIV* for cultures derived from E18, P0, and P10 rat cerebellum (Purkinje neuron per 500 mm^2^; E18: 13 ± 4; P0: 8 ± 1, P10: 1 ± 1; Fig. 1A).

Next, we plated a mixture of cerebellar cells and cells of the pons derived from E18, P0 or P10 rat tissue onto PDL-coated coverslips as an initial support cell layer. After culturing the support layer for 7 to 48 days, we plated another cell layer, a pure cerebellar cell layer, on top. We found that the tissue age of cells used to grow the support cell layer (E18 to P10) had no impact on the Purkinje neuron yield of the Purkinje neuron layer (data not shown). However, the in vitro age of the support layer influenced survival of neurons in a manner that depended on the tissue age used to grow the Purkinje neuron layer. The highest survival rate for E18-derived Purkinje neurons was observed when plated onto a support cell layer of 14 *DIV* (1153 ± 324); for P0-derived Purkinje neurons a support layer of 21 *DIV* (477 ± 136) was optimal; and for P10-derived Purkinje neurons, the optimal support layer age was 28 *DIV* (84 ± 4) (Fig. 1A). These findings indicate that the older the starting tissue of the Purkinje neuron layer is, the more mature the support layer must be to achieve a high survival rate of Purkinje neurons. We were not able to grow Purkinje neurons from more mature and adult tissue, such as postnatal day 14, 28, 60, and 180.

Furthermore, we found that the high survival rate in long-term Purkinje neuron culture could only be maintained when non-physiological pH fluctuations were avoided. The higher metabolic demand of the multilayer culture required a short cycle of media replacement. The optimized protocol involved 50% media replacement every 3.5 days for 6-well plates or every second day for 12- and 24-well plates.

### Dendritic Morphology of Purkinje Neurons: Importance of Paracrine Factors and Protein Kinase C Activity

Despite the improvement in Purkinje neuron survival resulting from multilayer culture, the dendritic morphology of the embryonic and early postnatal derived Purkinje neurons did not reflect that observed in vivo*.* E18- and P0-derived Purkinje neurons had fewer and shorter branches than those of P10 rats (Fig. 1B). Purkinje neuron survival and early dendritic tree development are highly dependent on paracrine factors such as progesterone, insulin, and insulin-like growth factor 1 (IGF1), which are secreted by other cells or self-produced by Purkinje neurons during the development of the cerebellum [23–25]. Addition of 40 µM progesterone to the growth medium led to increased branching of the dendritic trees in E18-derived Purkinje neurons (Fig. 1C) but had no impact on the branch structure of P0- and P10-derived Purkinje neurons (data not shown). The supplementation of only insulin or IGF1 did not improve the branch development but did stabilize the long-term growth of the other cerebellar cell types (data not shown). Therefore, insulin, IGF1 and progesterone had to be administered to all three Purkinje neuron cultures to archive a high survival of Purkinje neurons in long-term culture (data not shown).

We next tested the impact of inhibition of calcium-dependent protein kinase C (PKC) in the multilayer culture. PKC is known to be important for long-term anatomical maturation of the Purkinje neuron dendritic tree [26]. PKC inhibition using the chemical antagonist K252a considerably improved dendritic branching of Purkinje neurons in E18- and P0-derived Purkinje neuron layers (Fig. 1B and 1D). This effect was not seen in the P10-derived culture, however (Fig. 1D). Interestingly, PKC inhibition greatly improved the survival of P10-derived Purkinje neurons (by a factor of 28) in a concentration-dependent manner, and a concentration dependence was also seen for P0-derived Purkinje neurons (by a factor of 6; Fig. 1E). Inhibiting PKC activity had no effect on the survival rate of E18-derived Purkinje neurons (Fig. 1E).

Thus, to achieve a well-developed rat Purkinje neuron culture with a functional network the K252a concentration had to be adjusted to the used tissue age during the first 10 days in culture: K252a was used at 5 nM for E18-derived cultures, at 10 nM for P0-derived cultures, and at 25 nM for P10-derived cultures. At 10 *DIV*, the K252a concentration was set to 25 nM for all groups. At 22 *DIV*, the washout phase of K252a was begun, and the concentration of K252a dropped to zero at 28 *DIV* due to media replacement. At 28 *DIV*, the morphology of the Purkinje neurons closely resembled the in vivo morphology, and therefore, the IGF1 and progesterone concentrations were reduced by factors of 2.5 and 2, respectively, during subsequent culture.

### Cerebellar Network: Cells and Active Synapses

**Fig. 2:**
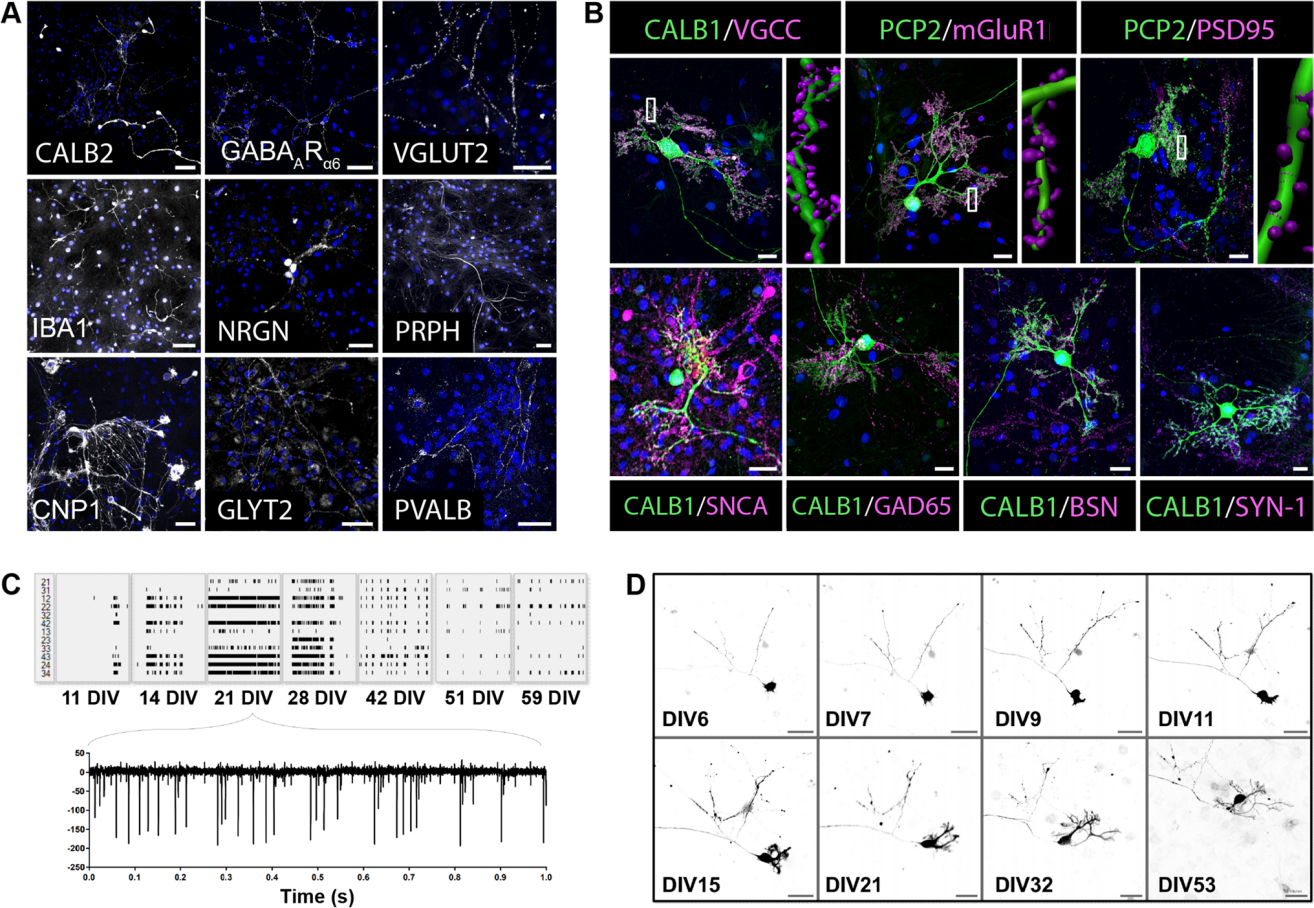
Purkinje neurons are active in the multilayer culture. **A** Representative immunohistochemical images of *DIV*28 cultures stained for the major cell types with calretinin (CALB2; unipolar brush cells), GABA_A_R_α6_ (granule cells), ionized calcium binding adaptor molecule 1 (IBA1; microglia), neurogranin (NRGN; Golgi cells), 2′,3′-cyclic-nucleotide 3′-phosphodiesterase (CNP1; oligodendrocytes), glycine transporter 2 (GLYT2; Lugaro cells), and parvalbumin (PVALB; stellate and basket cells). Fibres such as mossy and climbing were visualized with vesicular glutamate transporter 2 (VGLUT2) and peripherin (PRPH). DAPI was used as nuclear marker (blue). Scale bar: 50 µm. **B** Representative immunohistochemical images of mature Purkinje neurons at *DIV*28 stained with either calbindin (CALB1) or Purkinje cell specific protein 2 (PCP2) in green and post- or pre-synaptic biomarkers (magenta). IMARIS 3D reconstruction of the positive synapses of a chosen Purkinje neuron dendrite (indicated by rectangle) are shown to the right of images stained for post-synaptic markers VGCC, mGluR1, and PSD95. Pre-synaptic markers were α-synuclein (SNCA; a marker of glutamatergic synaptic terminals from granule cells/parallel fibers and unipolar brush cells), glutamate decarboxylase 65 (GAD65; a marker of axon terminals from stellate and basket cells), bassoon (BSN; a marker of the active zone of mossy fiber terminals and parallel fiber terminals between Golgi cells and granule cells and between basket cells and Purkinje neurons), and synapsin I (SYN-1; synaptic vesicle phosphoprotein of mature CNS synapses). DAPI used as nuclear marker (blue). Scale bar: 20 µm. **C** Multi-electrode array recorded spike patterns (10 s) at 11, 14, 21, 28, 42, 51, 59 *DIV* with a cut-out (1 s) at 21 *DIV* to follow Purkinje neuron maturity. **D** Representative live-cell imaging of an E18-derived Purkinje neuron expressing lentiviral-induced GFP at 6, 7, 9, 11, 15, 21, 32, and 53 *DIV*. Scale bar: 50 µm

To demonstrate that the Purkinje neuron multilayer culture system results in formation of functional synapses, we used immunostaining to identify cells and their pre- and post-synapses at 28 *DIV*. In addition to Purkinje neurons, the cultures contained neuronal cells such as GABA_A_R_α6_-positive granule neurons, neurogranin (NRGN)-positive Golgi, glycine transporter 2 (GLYT2)-positive Lugaro cells, calretinin (CALB2)-positive unipolar brush cells, parvalbumin (PVALB)-positive stellates, and PVALB-positive basket cells as well as non-neuronal cells such as GFAP-positive astrocytes (data not shown), 2′,3′-cyclic-nucleotide3′-phosphodiesterase 1 (CNP1)-positive oligodendrocytes, and ionized calcium binding adaptor molecule 1(IBA1)-positive microglia (Fig. 2A). We observed also fibre-like structures that were positive for vesicular glutamate transporter 2 (VGLUT2) and peripherin (PRPH) indicating mossy and climbing fibers (Fig. 2A).

Furthermore, we found that the cultured Purkinje neurons formed functional synapses that showed post-synaptic expression of voltage gated calcium channels (VGCCs), metabotropic glutamate receptor 1 (mGluR1), and post-synaptic density protein 95 (PSD95) (Fig. 2B, upper panel). Pre-synaptic contacts to the surrounding network were detected through synapses that were positive for glutamate-decarboxylase (GAD65), α-synuclein (SNCA), bassoon (BSN), and synapsin I (SYN1) (Fig. 2B, lower panel). The presence of these biomarkers indicates the maturity of both the Purkinje neurons and the surrounding network.

Next, we tested whether the visualized synaptic network is functional. In vivo, Purkinje neurons fire spontaneous action potentials at frequencies of around 40–50 Hz with a complex trimodal pattern of tonic firing, bursting, and silent modes that depend on the anatomy and functional maturity [27, 28]. We performed multi-electrode array recordings of E18-derived Purkinje neurons that were cultured in a 24-well multi-electrode array. Cultures grown in our Purkinje neuron multilayer culture media first showed spontaneous spike activity at 11 *DIV* (0.15 ± 0.03 Hz). The spike rate increased to 2.56 ± 0.59 Hz at 21 *DIV*. After 28 *DIV*, the spike activity became erratic with long periods of silence, but a frequency of 2.79 ± 0.55 Hz was maintained until 59 *DIV* (Fig. 2C). We observed both uniform and highly non-uniform spike intervals and trains with silent periods between bursts and spike frequencies of up to 140 Hz within the bursts. We also evaluated Purkinje neurons activity after exchanged into previously described organotypic brain slice culture media [22] at 28 *DIV*. These neurons did not show the erratic spike activity after 28 *DIV*. The organotypic brain slice culture media, which was developed to address the needs of a more mature neuronal network, stabilized the spike frequency at 6.35 ± 1.85 Hz through 63 *DIV* (data not shown).

### Cell Type-Specific Genetic Engineering

**Fig. 3:**
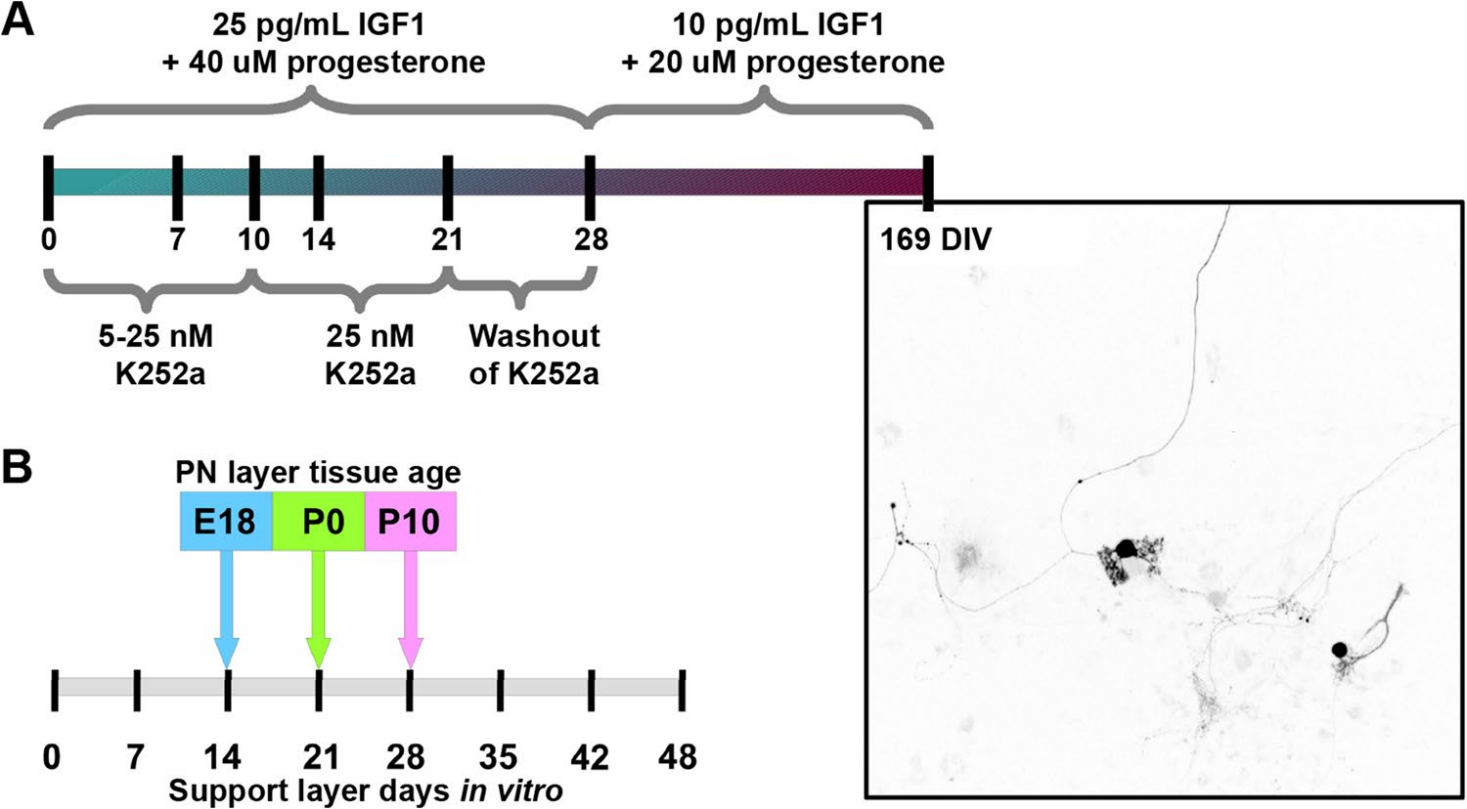
Optimal conditions for culture of rat Purkinje neurons. **A** Supplementation with IGF1 and progesterone induced a stable environment that resulted in high survival rates of Purkinje neurons, whereas PKC inhibition (with K252a) shaped dendritic tree development for E18 and P0 cultures or impacted survival for P10-derived culture. The K252a starting concentration depends on the tissue used to start the culture. During the washout phase (*DIV* 22–28), no K252a is added to the media. At 28 *DIV*, concentrations of IGF1 and progesterone are reduced. The protocol allows growth of a stable Purkinje neuron culture for up to 6 months (*DIV* 169) in 6- to 24-well formats. **B** The time point for placement of the Purkinje neuron layer depends on the age of tissue harvest. For E18, the highest survival was found when placed on a support layer of 14 *DIV*; for P0, the support layer should be 21 *DIV*; and for P10, the support layer should be 28 *DIV*

Cell type-specific genetic engineering is often an important approach in studding disease mechanisms. Therefore, we evaluate the potential of our Purkinje neuron model system for this approach by using lentiviral particles that transduce a viral vector for expression of green fluorescence protein (GFP) driven by the L7 promoter specifically into Purkinje neurons [16, 17]. The viral L7-GFP particles were added to dissociated Purkinje neurons on the day of seeding. Within 6 days, we observed Purkinje neurons expressing GFP (Fig. 2D). At 14 *DIV*, 61.5% of the cultured Purkinje neurons expressed GFP with minimal off-target expression (< 0.02%; data not shown). No alterations were seen in the dendritic structure due to thus endogenous protein expression. We observed a stable expression of GFP through 169 *DIV* (Fig. 3A). A high transfection rate was also achieved when the lentiviral particles were added to the culture at 14 *DIV* and 28 *DIV*; however, transfection efficiency and expression level fell progressively the later the genetic manipulation was implemented (data not shown). Live cell imaging of GFP-positive cultured Purkinje neurons showed very similar development to that seen in vivo which is characterized by three phases [29, 30]. In our E18 culture, at *DIV* 0 to 7, we observed the 1^st^ phase, the fusion phase that happens from embryonic day 17 to postnatal day 5; at *DIV* 7 to 9, we saw the 2^nd^ phase, the phase of stellate cells with disoriented dendrites that occurs at postnatal day 5 to 7; and from *DIV* 9 to 23, we observed the 3^rd^ phase, the phase of orientation and flattering of the dendritic tree that starts at postnatal day 7 and continues for 14 days until a Purkinje neuron reaches maturity (Fig. 2D).

## Discussion

To investigate the mechanisms underlying neurodegenerative disorders, good model systems are vital. Rat and mouse models are commonly used in biomedical research [6, 8–12], and several protocols for generating cerebellar cultures from mice, including some with healthy Purkinje neurons, have been published [3, 5]. Rats are more physiologically, genetically, and morphologically similar to humans than are mice, and therefore, rat models mimic many human neurodegenerative disease mechanisms better than mouse models do [6–9, 22, 31, 32]. Furthermore, rats have a bigger size and higher stress tolerance than mice, and therefore, operative approaches are easier with low environmental impact [6].

Rat cerebellar cell cultures have been of limited utility, however [1, 2, 4]. In previously reported systems, the long-term Purkinje neuron survival and dendritic development in culture were very poor when using postnatal rat tissue [1]; therefore, only embryonic tissue has been used [1, 2, 4]. Furthermore, the neuronal activity of long-term cultured Purkinje neurons has not been demonstrated [2, 4]. Here we present an ex vivo culture protocol that not only leads to mature and synaptically active rat Purkinje neurons in a complex and robust long-term culture system but also allows for maximal experimental flexibility. The combined use of a support layer and time-dependent addition of hormones, paracrine factors, and biochemical activity regulators such as progesterone, insulin, IGF1, and K252a gave ideal conditions to grow a balanced cerebellar network from either embryonic or postnatal tissue (Fig. 3).

We showed that four factors are important for long-term survival as well as well-developed Purkinje neurons: (1) the support layer, (2) pH stability, (3) co-factor supplements, and (4) starting tissue age. The support layer affects the pH stability and also provides important cell-to-cell contacts that drive the release of paracrine factors. We found that fluctuation of the pH into the basic range (> 7.5), often seen in monolayer culture, reduced the long-term survival of Purkinje neurons. Fluctuation into the acid range (< 6.5) was better tolerated but indicated a high metabolic demand and therefore the need for a shorter feeding cycle. The fluctuations of the pH are prevented in our protocol by preparing media immediately before use and by feeding the support layer 24 h before plating the Purkinje neuron layer.

Survival and dendritic tree development of the Purkinje neurons are highly dependent on secreted factors and cell–cell interactions. Paracrine factors such as progesterone, insulin, and IGF1 are released by the cell network in an age-dependent manner to ensure Purkinje neuron development and maturity as well as the stability of the surrounding neuronal network [23–25]. This age-dependent release was clearly seen in our model, as we observed that that the Purkinje neuron culture derived from postnatal tissue needed a more mature support cell layer than embryonic-derived Purkinje neurons. Addition of progesterone improved the dendritic tree branching of E18-derived Purkinje neuron but did not alter the branching in the two postnatal cultures.

Calcium homeostasis of the neuronal network is also critical during development [26, 29, 33, 34]. Neuronal dendrites are generated during development by a series of processes involving extension and retraction of dendritic branches and subsequent stabilization of existing dendrites through synaptic connections and neuronal calcium homeostasis [34]. Calcium-dependent PKC subtypes, activated by synaptic inputs from parallel fibres (granule cells) through mGluR1/4, trigger functional changes as well as long-term anatomical maturation of the Purkinje neuron dendritic tree during cerebellar development [26, 29]. Minimizing the PKC activity by the application of K252a improved the dendritic tree development of Purkinje neurons derived from E18 and P0 tissue and considerably improved the survival of Purkinje neurons that were derived from P10 tissue. These results are indicative of biphasic action of PKC during Purkinje neuron development. Furthermore, analyses of the Purkinje neuron culture under modulated PKC activity revealed well-developed synapses that were positive for P/Q-VGCC and mGluR1, markers indicative of maturity. Electrophysiological recordings also indicated that the cultured Purkinje neurons matured throughout the cultivation period. The spontaneous activity of the Purkinje neurons was very similar to activities detected in vivo recordings: Purkinje neurons fire spontaneous action potentials with a complex trimodal pattern of tonic firing, bursting, and silent modes [27, 28]. However, the fire frequencies of spontaneous action potentials in our multilayer culture was only one-tenth of the frequency (40–50 Hz) that was recorded in vivo [27, 28]*.* As the neuronal network of our model system is not as well organized as the in vivo network and consists of only an approximately thickness of 50 µm, the lower frequency was not unexpected.

We also demonstrated that the Purkinje neurons in our model system could be genetically edited. The GFP-positive Purkinje neurons in our E18 culture system developed similarly to stages previously characterised in vivo [29, 30, 34]. We observed the fusion phase, the phase of stellate cells with disoriented dendrites, and the orientation and flattering of the dendritic tree. At 15 *DIV*, the E18-derived Purkinje neurons had a very complex dendritic arbour structure similar to that observed in P12 rats. We were unable to culture functional Purkinje neurons from rat tissue older than P10 under the optimized conditions. All attempts with tissue of age P14, P28, P60, and P180 failed. The live-cell imaging of the GFP tagged E18 Purkinje neurons at *DIV*15, which is equivalent to P12, showed that the dendritic arbore consisted of increasing numbers of fine branches that have entered the 3rd phase of development [29, 30, 34]. Therefore, neurons older then P10 cannot be separated without breaking the fine branches and killing the cell.

## Conclusion

In summary, the rat Purkinje neurons grown in a multilayer long-term culture were (1) positive for all tested synaptic markers; (2) electrically active; and (3) responsive to genetic manipulation. This demonstrates that the culture protocol is robust and versatile and can thus be used in a wide variety of cerebellar studies. For example, this culture model should prove useful as a high-throughput screening tool to investigate disease mechanisms as well as for testing of potential drugs.

## Data Availability

The datasets generated during and/or analysed during the current study are available from the corresponding author on request*.*

## Abbreviations

E18: Embryonic day 18
DIV: Days in vitro
FBS: Foetal bovine serum
GAD65: Glutamate-decarboxylase 65
GFP: Green fluorescence protein
GLYT2: Glycine transporter 2
IGF1: Insulin-like growth factor 1
mGluR1: Metabotropic glutamate receptor 1
P0: Postnatal day 0
P10: Postnatal day 10
PKC: Protein kinase C
PDL: Poly-d-lysine
PSD95: Post-synaptic density protein 95
VGCC: Voltage-gated calcium channels
